# Thirty years of coral reef change in relation to coastal construction and increased sedimentation at Pelekane Bay, Hawaiʻi

**DOI:** 10.7717/peerj.300

**Published:** 2014-03-13

**Authors:** Yuko Stender, Paul L. Jokiel, Kuʻulei S. Rodgers

**Affiliations:** 1 Hawaiʻi Institute of Marine Biology, University of Hawaiʻi, Kaneohe, Hawaiʻi, USA; 2 Department of Geography, University of Hawaiʻi at Mānoa, Honolulu, USA

**Keywords:** Coral reefs, Land-based impacts, Hawaiʻi, Long-term monitoring, Sedimentation

## Abstract

Coral reefs are being critically impacted by anthropogenic processes throughout the world. Long term monitoring is essential to the understanding of coral reef response to human impacts and the effectiveness of corrective management efforts. Here we reevaluated a valuable coral reef baseline established in Pelekane Bay, Hawaiʻi during 1976 and subsequently resurveyed in 1996. During this time interval substantial impacts occurred followed by extensive corrective measures. Coral and fish communities showed dramatic declines from 1977 to 1996 due to massive harbor construction and suboptimal land management practices on the watershed. More recently, corrective measures in the form of watershed stabilization and fishing regulations have been implemented. Consequently our 2012 survey reveals that coral cover since 1996 has increased slightly accompanied by a significant increase in fish abundance, diversity, and evenness. This improvement can be attributed to lower fishing pressure since 1996 due to reduced shoreline access, tighter fishing regulations and increased monitoring of legal and illegal fishing activities. Stabilization of the coral community can be attributed partially to reduced sedimentation resulting from watershed restoration that included installation of sediment check dams, control of feral ungulates, controlled grazing and replanting of native vegetation. Insights into the mechanism that removes sediment from reefs was provided by a major storm event and a tsunami that remobilized and flushed out sediment deposits. The increase in herbivorous fishes probably played a role in reducing algal competition in favor of corals. The data suggest that the precipitous reef decline in this area has been arrested and offers support for the corrective actions previously undertaken.

## Introduction

Coral reefs have been impacted by anthropogenic processes on a global scale (Bryant et al., 1998; Richmond et al., 2007; Halpern et al., 2008; Wilkinson, 2008). Direct impacts of global climate change on coral reefs is a great concern (Hughes et al., 2003; Hoegh-Guldberg, 2011), but indirect local effects such as altered hydrological processes (Fletcher, 2010) also impose land-based threats to coral reefs. Deforestation, uncontrolled grazing and other destructive practices accelerate erosion with a concomitant increase in delivery of terrigenous sediments, associated nutrients and pollutants to coral reefs (Syvitski et al., 2005; Maina et al., 2013). Habitat degradation and or loss from anthropogenic activity impairs the ability of corals to recover from perturbations (Wolanski et al., 2003; Richmond et al., 2007). Sedimentation has long been known to be one of the major threats to coral reefs worldwide (Johannes, 1975), and appears to be the main stressor in Pelekane Bay. Sediments interfere with ecological functions (Rogers, 1990; Richmond, 1993; Fabricius, 2005; Johansen & Jones, 2013). Quantitative and comprehensive studies substantiate the negative effects of sedimentation on coral growth, morphology, and development at all coral life stages (Grigg & Birkeland, 1997; Te, 2001). Extensive research has been published on lethal and sublethal effects of sediment including reduced reproductive output, lower recruitment rates (Birkeland, 1977; Rogers, 1990), decreased calcification (Randall & Birkeland, 1978), morphological changes (Dustan, 1975; Brown, Howard & Le Tissier, 1986), metabolic changes (Te, 2001), behavioral alterations (Brown & Howard, 1985; Rogers, 1990), and increases in diseases and bleaching (Brown & Howard, 1985). Researchers have identified detrimental impacts to corals from toxins associated with sediment such as chemicals and heavy metals. These toxins adsorb onto sediment and even at low concentrations can produce adverse secondary effects in corals (Glynn et al., 1986; Glynn et al., 1989).

**Figure 1:**
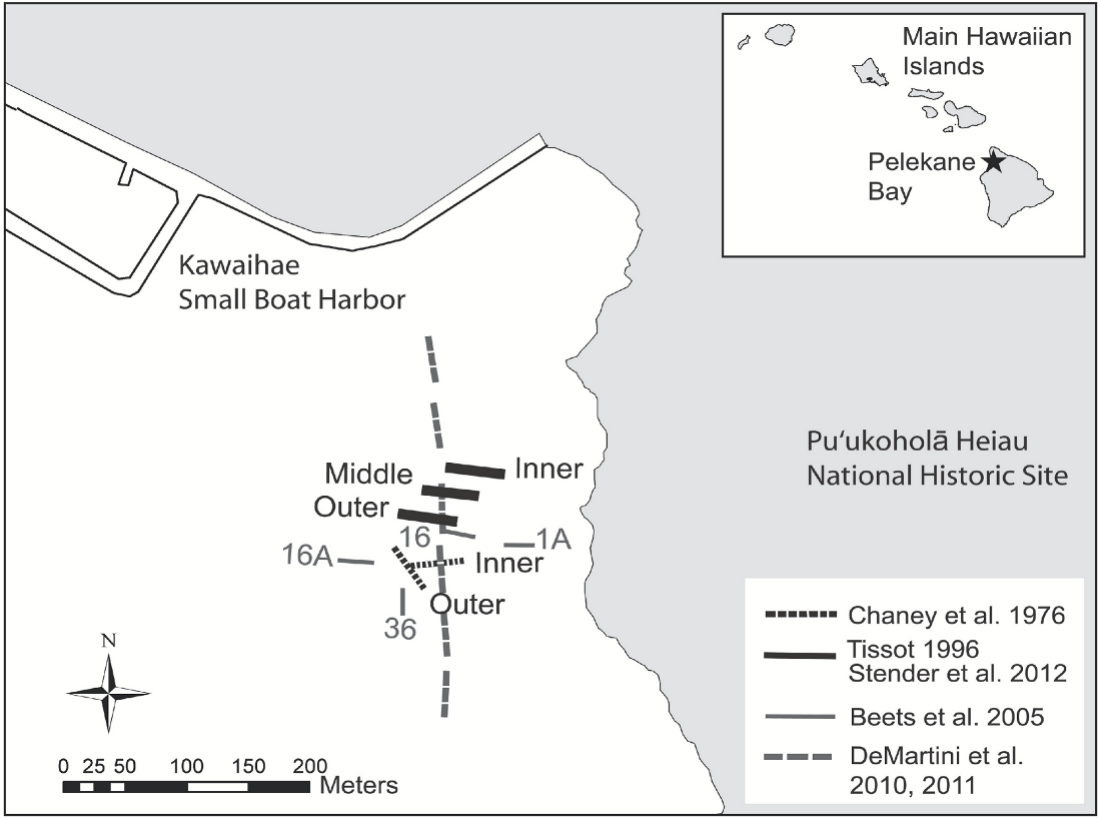
Map of historical and present survey locations. Map of historical and present survey locations, Pelekane Bay, Hawaiʻi with adjacent Kawaihae harbor and watershed (GIS data source: Hawaiʻi State GIS).

Pelekane Bay, located on the south Kohala Coast of the island of Hawaiʻi, has historically been subjected to major alterations (Fig. 1). Since the early 1800s there have been extensive large-scale modifications of the Kawaihae watershed that drains into the bay (Greene, 1993). Introduction of cattle by Captain Vancouver in 1793 and the harvest of sandalwood (‘*iliahi*) from the upper reaches of the Kawaihae watershed decimated this once lush forest and caused increasing sedimentation and alteration of natural water flow patterns. Early historical accounts on effects of deforestation and grazing describe a nearly barren landscape with a cessation of perennial streams by 1830 (Kelly, 1974). Subsequent impacts continued with dredge and fill operations that removed a large fringing reef to create the adjacent Kawaihae Harbor in 1959 (Fig. 1). Long-shore currents were disrupted by construction of breakwaters and a large filled area to the north of Pelekane Bay. Massive explosive charges were used by the US Army’s Nuclear Cratering Group (Project Tugboat) to create a small boat harbor north of Pelekane Bay during 1969 to 1970. The blasting deposited extensive coral silt and rubble on the reef and reduced ocean circulation in Pelekane Bay (Day, 1972). Several studies described the Pelekane Bay coral reef communities during this time (Cheney, Hemmes & Nolan, 1977; Tissot, 1998). The Environmental Protection Agency (EPA) currently lists Pelekane Bay as an “impaired waterbody” due to sedimentation. In addition, the adjacent watershed has been identified by the Hawaiʻi Coral Reef Strategy (The Kohala Center, 2011) as one of the watersheds in most critical need of restoration. Diverse research projects have been conducted in the past decade by numerous organizations in the Pelekane region (e.g., Hoover & Gold, 2006; Cochran, Gibbs & Logan, 2007; Group 70 International, 2007; Beets, Brown & Friedlander, 2010; Thornberry-Ehrlich, 2011; The Kohala Center, 2011; Minton et al., 2011; Storlazzi et al., 2013; DeMartini et al., 2013).

Evaluation of watershed impacts on a coral reef requires quantitative measurement of the biological changes in the area that receives the runoff. A baseline for marine vertebrates and invertebrates (Cheney, Hemmes & Nolan, 1977) and marine algae (Ball, 1977) was established over three decades ago in Pelekane Bay. These surveys allowed Tissot (1998) to describe dramatic declines in biota that occurred between 1976 and 1996. The objective of the present study was to re-survey the fish and benthic communities surveyed by Tissot (1998) in order to document changes in this area. Detection of long term changes is critical for identifying issues and developing solutions (Jokiel et al., 2004).

## Materials and Methods

**Figure 2:**
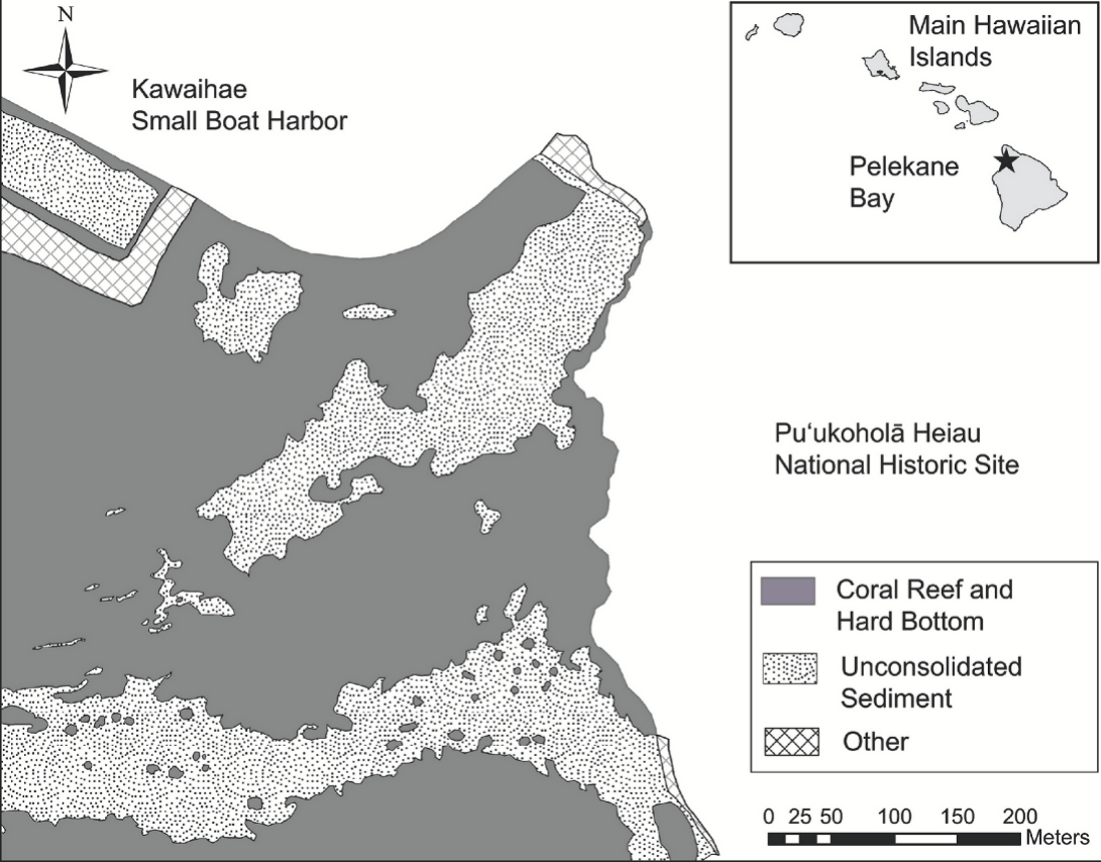
Benthic habitat map of the study area. Map of benthic habitat, Pelekane Bay, Hawaiʻi with adjacent Kawaihae harbor and watershed (GIS data source: Cochran, Gibbs & Logan, 2007, Hawaiʻi State GIS).

Ecological surveys were conducted between 18 and 23 June, 2012. The map of Pelekane Bay in Tissot (1998) was processed in ArcGIS in order to determine exact transect locations. The map was geo-referenced using polynomial coefficients derived from a set of small, well-defined landscape features (Richards & Jia, 2006) with known geographic coordinates on a satellite image. Beginning and ending locations of each transect were established using a Garmin GPSMAP 78sc. Three parallel 50 m transects were reestablished (Fig. 1) following the descriptions and map in Tissot (1998). All three transects (Fig. 1) fall into the coral reef and hard substrate category described by Cochran, Gibbs & Logan (2007) based on the NOAA habitat maps (Fig. 2).

Relative abundance and composition of benthic organisms and substrate were quantified using *in situ* photographs. Approximately 100 high resolution digital images were taken along each of the three 50 m transect lines on 18 June, 2012 using an Olympus 5050 zoom digital camera with an Olympus PT050 underwater housing. An aluminum monopod frame positioned the camera vertically at 0.7 m above the substrate to provide a standardized 0.35 m^2^ image area. A 6-cm bar on the monopod base served as the reference scale in each image.

The software program PhotoGrid (Bird, 2001) was used to quantify percent cover of benthic organisms including individual coral species and higher taxonomic algal groups (e.g., coralline algae, turf, macro, etc.) and abiotic substrate. For each 50 m transect, 100 images were selected and 25 random points were displayed onto each image for analyses.

Rugosity measurements of topographical relief were conducted along each transect. A 15 m chain marked at 1 m intervals with 1.3 cm links was draped along the length of each transect following the contours of the benthos. An index of rugosity was calculated using the ratio of the reef contour distance as measured by chain length to the linear horizontal distance (McCormick, 1994).

Repeated fish surveys were initiated at approximately the same time of day during the survey period to reduce temporal variability. A visual belt transect approach was employed (Brock, 1954) with numerical abundance, species, and total length of fishes recorded (Brown et al., 2003). A diver swam along the three 50 m × 4 m transects (200 m^2^) at >1 h intervals between surveys. Ten replicates of each of the three transects were conducted (*n* = 30) within the six day survey period.

Water quality was measured in order to establish the relative conditions along a gradient from the stream mouth to open waters during the survey period. Data were collected on 23 June 2012, using a multi-parameter water quality meter (YSI 6920 V2 SONDE). Water quality measurements included temperature (°C), pH, salinity (ppt), and turbidity (NTU). Subsequent to the fish survey, a diver swam twice along each transect with the SONDE to take measurements at five second intervals. Time at the beginning and ending positions of transects was determined using a watch synchronized with the SONDE to verify data corresponding to each transect.

Statistical analysis was conducted using Minitab 15 (Minitab, Inc., 2007) to evaluate differences in abundance of reef fishes among years. Mean density per 100 m^2^ was calculated by species, families, and feeding guilds and ln(*x* + 1) transformed to address assumption of normality and homoscedasticity (Zar, 1999). Overall effects of year and transect on variations in fish abundance were appraised for 1996 and 2012 data using two-way analysis of variance (ANOVA) with subsequent Tukey’s HSD multiple comparisons. Each species was independently tested using one-way ANOVA and post-hoc multiple comparisons for all years. Similarly, each of the major family groups and feeding guilds were independently tested by year and followed by post-hoc Tukey’s HSD comparison for all years. Paired *t*-tests and percent change were used to compare temporal changes in coral cover and composition between years. The Shannon-Weiner diversity index was used to calculate fish diversity. Standard errors of the means (mean ± s.e.) were reported with mean density of fish to describe the measure of the uncertainty.

## Results

### Benthic surveys and environmental conditions

On 23 June 2012, the inner and middle transects were characterized by similar water quality but with slightly higher temperature, lower pH, lower salinity, and greater turbidity than on the outer transect. Turbidity was highest at the inner transect followed by the middle and outer transects as distance from the stream source increased. Conversely, salinity and rugosity were lowest along the inner transect and increasing with distance on the outer transects (Table 1).

**Table 1:** Table of descriptive statistics for water quality parameters. Means of temperature, pH, salinity, and turbidity were measured at the inner (*n* = 137), middle (*n* = 133), and outer transects (*n* = 165). Coefficient of Variation is indicated in parentheses.

Variables	Inner	Middle	Outer
Temperature (°C)	27.0 (0.1%)	26.9 (0.2%)	26.4 (0.5%)
pH	8.10 (0.1%)	8.10 (0.0%)	8.13 (0.1%)
Salinity (‰)	34.7 (0.2%)	34.8 (0.2%)	35.1 (0.3%)
Turbidity (NTU)	1.8 (15.7%)	1.5 (18.8%)	0.8 (34.5%)
Rugosity (*n* = 5)	1.54 (9.3%)	1.67 (5.9%)	1.91 (6.8%)

There was a substantial drop in overall coral cover between 1976 (44%) and 1996. The change between 1996 (5.5%) and 2012 (6.6%) was not statistically significant (Table 2).

**Table 2:** Table of overall mean coral cover across survey years. Change in total coral cover at Pelekane Bay between 1976 and 2012. One standard errors of the mean are indicated by ± s.e.

Survey year	Month	Author	Mean cover (%) ± s.e.
1976	April	Cheney, Hemmes & Nolan (1977)	43.45 ± 2.45
1996	January–April	Tissot (1998)	5.50 ± 2.26
2012	June	Stender et al. (2014)	6.58 ± 2.15

Coral species richness declined from 1976 (9 species) to 1996 (5 species, 44% decline) and subsequently increased in the 2012 surveys (8 species, 60% increase since 1996, Table 3). Species composition also shifted. Five of the species found on transects in 1976 were not present in 1996. Three species recorded in 2012 were not found in 1996 (Table 3). Statistically significant differences were not found in coral species distribution between these years due to high variability resulting from patchy distribution. The coral species, *Porites lobata* was dominant in 1996 with 3.9% cover and again in 2012 with 4.0% cover. *Porites compressa* increased substantially since 1996 while other less dominant species remained relatively constant (Table 3). *Montipora capitata* (0.4% cover) showed a marked decline since 1976 (7.2% cover). The inner transect (3.3% cover) had the lowest coral cover followed by the middle (5.8% cover), and the outer transect (10.6% cover). The area covered by silt showed a consistent decline from 1976 (41.0%) to 1996 (30.5%) to 2012 (24.4%).

## Fish Surveys: Species

A shift in species composition was detected between the three surveys (Table 4). Overall percent similarity in the fish community between 1996 and 2012 was 28.5% compared to 27.6% between 1976 and 2012. Twenty species recorded on transects in 2012 were not noted in 1996 and eighteen species documented in 1996 were not recorded in 2012. In the baseline 1976 surveys there were seven species not common to the subsequent 1996 and 2012 surveys. Statistically significant increases occurred in the abundance of five species between 1976 and 2012. These species included *Acanthurus nigrofuscus* (6.6 fish⋅100 m^−2^; *F*_2,5_ = 6.31, *p* = 0.043), *Abudefduf abdominalis* (2.7 fish⋅100 m^−2^; *F*_2,5_ = 6.58, *p* = 0.040), *Scarus psittacus* (2.4 fish⋅100 m^−2^; *F*_2,5_ = 7.21, *p* = 0.034), Gobiidae spp. (1.2 fish⋅100 m^−2^; *F*_2,5_ = 16.53, *p* = 0.006), and *Acanthurus blochii* (0.3 fish⋅100 m^−2^; *F*_2,5_ = 7.19, *p* = 0.034), while significant decreases were observed for *Stegastes marginatus* (0.2 fish⋅100 m^−2^; *F*_2,5_ = 28.11, *p* = 0.002) and *Ctenochaetus strigosus* (0.1 fish⋅100 m^−2^; *F*_2,5_ = 17.53, *p* = 0.006). Although the statistical significance of the mean abundance of *Thallasoma duperrey* was marginal (*F*_1,4_ = 5.25, *p* = 0.084), increase in its abundance was substantial from 1.4 fish⋅100 m^−2^ in 1996 to 4.8 fish⋅100 m^−2^ in 2012, more than the triple abundance of 1996. Similarly an increase in the abundance of *Chlorurus spilurus* was not statistically significant. However, it was numerically more abundant in 2012 (7.2 fish⋅100 m^−2^) than in 1996 (0 fish⋅100 m^−2^) or in 1976 (2.9 fish⋅100 m^−2^). While *Mulloidichthys flavolineatus* ranked as the most abundant fish in 1976, there was no statistical difference among survey years. The significant increase in the abundance of *Chaetodon lunula* reported between 1976 (0.0 fish⋅100 m^−2^) and 1996 (0.2 fish⋅100 m^−2^) did not occur in 2012 (0.2 fish⋅100 m^−2^). Observed declines occurred in juvenile Scarus spp. (4.1 fish⋅100 m^−2^; *F*_2,5_ = 128.77, *p* < 0.000), *Acanthurus nigroris* (0.6 fish⋅100 m^−2^; *F*_2,5_ = 31.68, *p* = 0.001), *Porphyreus cyclostomus* (0.2 fish⋅100 m^−2^; *F*_2,5_ = 56.16, *p* < 0.001), and *Chaetodon auriga* (0.2 fish⋅100 m^−2^; *F*_2,5_ = 12.87, *p* = 0.011) since 1996. Numerical declines since 1976 include *Chromis ovalis* (5.9 fish⋅100 m^−2^) and *Scarus dubius* (0.3 fish⋅100 m^−2^). A subset of additional data acquired by Beets et al. during 2005 (Beets, Brown & Friedlander, 2010) was reanalyzed using the four transects (1A, 16A, 16, and 36) in close proximity to the Cheney, Hemmes & Nolan (1977) and Tissot (1998) transects (Fig. 1). Four of the top five species in abundance found in 2005 were also found in the present study (*C. spilurus* 31 fish⋅100 m^−2^, *A. nigrofuscus* 7.4 fish⋅100 m^−2^, *S. psittacus* 4.0 fish⋅100 m^−2^, juvenile Scarids 3.2 fish⋅100 m^−2^, and *T. duperrey* 2.0 fish⋅100 m^−2^) with slight differences in rank order (Table 4).

**Table 3:** Table of coral cover by species across years. Coral cover by species (%). Richness in parentheses.

Species name	1976 (9)	1996 (5)	2012 (8)
*Cyphastrea ocellina*	0.85	0	0
*Leptastrea bottae*	0.9	0	0
*Montipora patula*	3.8	0	0.11
*M. capitata (verrucosa)*	7.15	0.6	0.44
*Pavona varians*	0.85	0	0.07
*P. duerdeni*	0	0	0.12
*Pocillopora damicornis*	0	0.8	0.12
*P. meandrina*	3.45	0.7	0.04
*Porites compressa*	15.9	0.7	2.06
*P. lobata*	11.05	3.9	3.95
*Porites sp.*	3.7	0	0

The fish community in 2012 shows higher species richness, overall Shannon-Weiner H′ diversity, and mean fish density as compared to previous surveys in 1976 and 1996 (Table 5). A marked difference in the mean density of fishes (per 100 m^2^) between the inner (0.29), middle (0.77), and outer transects (1.46) was found. Species richness and diversity (H′) was highest at the outer transect (35 species, H′ = 2.41) as compared to the inner (26 species, H′ = 2.29) and middle (25 species, Diversity = 2.39) transects, while similar evenness was calculated between the inner (0.70), middle (0.73), and outer (0.68) transects. A two-way ANOVA including year, transects, and interaction between years 1996 and 2012 with transects as predictors, was highly significant (${R}_{a d j.}^{2}=0.45$, *F*_5,50_ = 10.07, *p* < 0.000). Overall mean abundance in 2012 was statistically higher than in 1996 (*F*_1,50_ = 5.41, *p* = 0.024). It was influenced mainly by the outer transect (*p* < 0.000). There were also statistically significant effects of transect (*F*_1,50_ = 11.74, *p* < 0.000) and interaction between year and transect (*F*_1,50_ = 9.55, *p* < 0.000). Greater fish abundance was influenced mainly by the middle (*t* = 2.83, *p* = 0.018) and outer transects (*t* = 4.82, *p* < 0.000).

**Table 4:** Table of abundant top five species by year. Comparison of most abundant fish species in rank order among surveys.

	1976 (Chaney et al.)	1996 (Tissot)	2012 (Stender et al.)
1	Mulloidichthys samoensis^*^​	Juvenile *Scarus spp.*	*Acanthurus nigrofuscus*
2	*Chromis ovalis*	*Ctenochaetus strigosus*	*Chlorurus spilurus*
3	Scarus sordidus^**^​	*Gomphosus varius*	*Thalassoma duperrey*
4	*Thalassoma duperrey*	*Thalassoma duperrey*	*Scarus psittacus*
5	*Abudefduf abdominalis*	*Acanthurus triostegus*	*Abudefduf abdominalis*

**Notes.**

* Currently accepted name is *Mulloidichthys flavolineatus*.

** Currently accepted name is *Chlorurus spilurus*.

**Table 5:** Table of overall fish assemblage by years. Comparison of fish assemblage characteristics among surveys.

	1976 (Chaney et al.)	1996 (Tissot)	2012 (Stender et al.)
Mean number of fish⋅100 m^−2^	27.9	18.1	34.5
Species richness	35	39	41
Diversity (Shannon–Weiner )	1.07	1.17	2.46
Evenness	0.69	0.73	0.66

## Families

**Figure 3:**
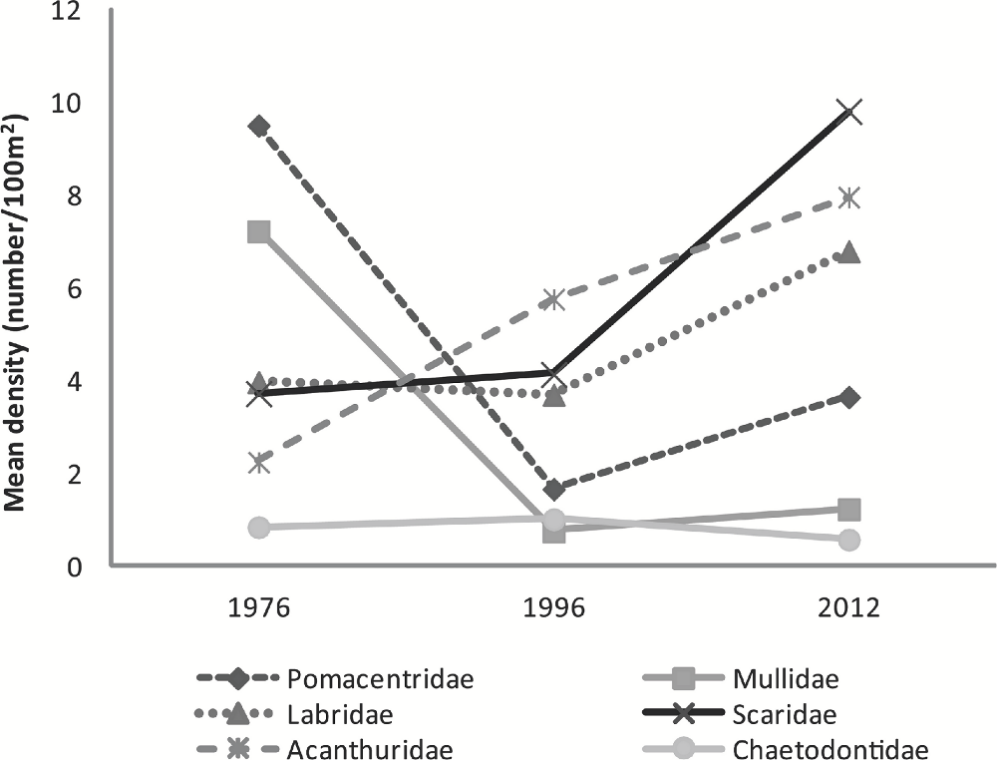
Graph of mean fish density by families across years. Mean abundance of major fish families across survey years.

Although substantial percent increases were found in the abundance of major family groups between 1996 and 2012 (Scaridae 137%, Pomacentridae 119%, Labridae 84%, Mullidae 63%, and Acanthuridae 38%) a statistically significant difference was found only in the abundance of Pomacentridae among years (*F*_2,5_ = 10.36, *p* = 0.017) with number of species within this family significantly declining between 1976 (9.5 ± 0.4 fish⋅100 m^−2^) and 1996 (1.7 ± 0.3 fish⋅100 m^−2^; *t* = −4.55, *p* = 0.014) and increasing in the 2012 study (3.7 ± 1.1 fish⋅100 m^−2^) (Fig. 3).

## Feeding Guilds

**Figure 4:**
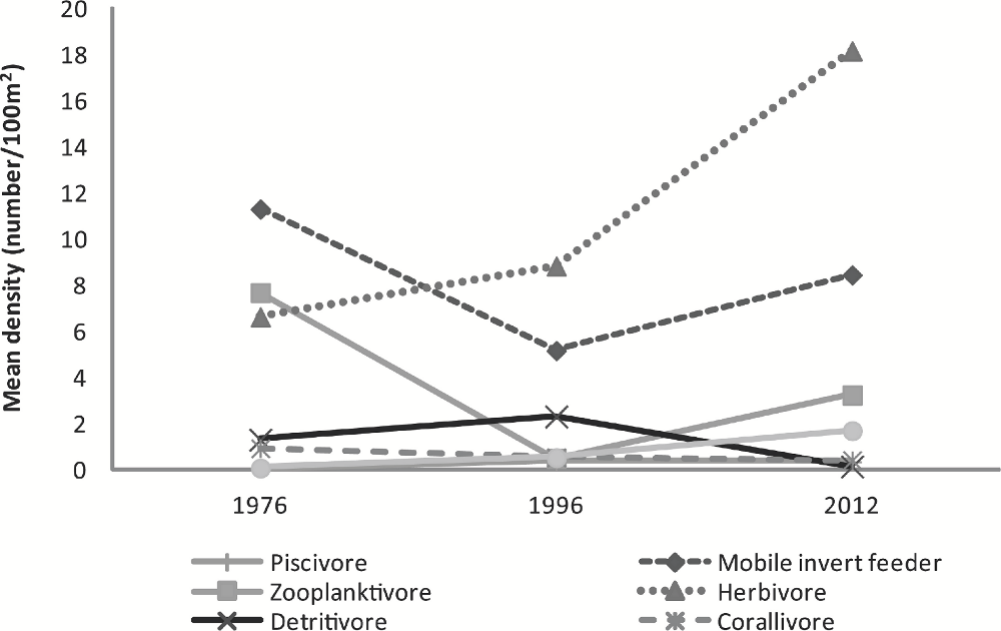
Graph of mean fish density by feeding guilds across years. Mean abundance of feeding guilds across survey years.

The mean abundance of herbivorous species has increased from 1976 (6.6 ± 3.4 fish⋅100 m^−2^) to 1996 (8.8 ± 0.5 fish⋅100 m^−2^) to 2012 (18.2 ± 8.2 fish⋅100 m^−2^) a 51% and 174% respective increase between years. Mobile invertebrate feeders (*F*_2,5_ = 28.85, *p* = 0.002), zooplanktivores (*F*_2,5_ = 13.16, *p* = 0.01), and detritivores (*F*_2,5_ = 18.87, *p* = 0.005) greatly varied among years. Sessile invertebrate feeders became significantly more abundant in 2012 (1.7 ± 0.3 fish⋅100 m^−2^) compared to 1976 (0.1 ± 0.03 fish⋅100 m^−2^; *t* = 7.32, *p* = 0.002) and 1996 (0.5 ± 0.1 fish⋅100 m^−2^; *t* = 5.08, *p* = 0.009). The decline in zooplanktivores was significant between 1976 (7.7 ± 0.1 fish⋅100 m^−2^) and 1996 (0.5 ± 0.4 fish⋅100 m^−2^; *t* = −5.02, *p* = 0.009), but marginally increased by 85% in 2012 (3.2 ± 1.1 fish⋅100 m^−2^; *t* = 3.17, *p* = 0.055). In contrast, detritivores were less abundant in 2012 (0.1 ± 0.1 fish⋅100 m^−2^) than in both 1976 (1.3 ± 0.6 fish⋅100 m^−2^; *t* = −3.45, *p* = 0.041) and in 1996 (2.3 ± 0.3 fish⋅100 m^−2^; *t* = −6.09, *p* = 0.004). *Ctenochaetus strigosus* was the only species comprising the detritivore feeding guild which decreased considerably over the 36 year period since the original surveys. Slight decline in corallivore was observed. Piscivores remained low across the surveys (Fig. 4).

## Discussion

Fish assemblage abundance, richness, and diversity in Pelekane Bay have improved over the past 16 years following a severe decline between 1976 and 1996. Our data also shows an increased abundance of herbivores. This pattern agrees with results of a 2005 survey by U.S. National Park Service Inventory and Monitoring Program (Beets, Brown & Friedlander, 2010). Species composition has shifted relative to the 1976 survey but remains similar to that observed in 2005. Results of the present survey are in agreement with the findings of DeMartini et al. (2013) who demonstrated a significant positive effect of improved habitat (lower sediment accumulation and greater availability of branching corals) on the density of juvenile parrotfishes. The same pattern of increasing fish abundance along a gradient of improving habitat was shown in our study as well as the study by Beets, Brown & Friedlander (2010).

The 2012 study showed stabilization and perhaps a slight increase in coral cover since 1996 following a substantial reduction between 1976 and 1996. The increase in herbivorous fishes has likely helped the coral population by reducing algal competition in favor of corals. Moreover, recent episodic large wave events demonstrate that natural processes remove accumulated sediment deposits on coral reefs. The November 2010 flash flood introduced a high sediment load into the bay, but the residence time of the sediment was short due to a subsequent large wave event in January which transported the sediment into deep water offshore (Storlazzi et al., 2013). This was followed by the March 2011 tsunami that re-suspended and removed a great deal of sediment from the reef (DeMartini et al., 2013). Such events may remediate sediment impacts on the benthic community and improve inshore habitat quality over time.

Since 1996 there have been substantial changes at Pelekane Bay that may explain the increases in fish populations. A public county road that formerly ran along the coastline was realigned at a higher elevation in 1996 in order to restore the shoreline to conditions that existed at the time when the historic Pu‘ukoholā temple was dedicated. Removal of the road limited shoreline accessibility. New rules restricted camping to Spencer Beach Park at the south end of Pelekane Bay which resulted in lowered fishing pressure in the study area. In addition, a new NPS visitor information center was built in 2007. The visitor center is located close to the bay with an overlook complete with telescopes that allows for constant observation of the reefs by visitors and rangers. Rangers now conduct patrols along the shoreline as part of their duty. Access to Pelekane Bay from the harbor area to the north was further restricted in 2011 due to increased harbor security under the Homeland Security Program at Kawaihae Harbor following the terrorist attack of Sept. 11, 2001.

The establishment of nearby marine protected areas designated by the State of Hawaiʻi in 1998 may also have contributed to the increase in fish populations. In select regions, the West Hawaiʻi Fisheries Management Areas (FMAs) and Fisheries Replenishment Areas (FRAs) were designed to limit high take methods of fishing, create fish reserves. Marine protected areas (MPAs) act as fish refuges with research demonstrating an increase in the number and size and connectivity within and between reserves (Friedlander et al., 2010). Areas adjacent to reserves benefit as fishes move in and out of the area and “spill-over” into nearby regions (Birkeland & Friedlander, 2001). The “spill-over” effect was particularly significant for resource fishes including parrotfishes in Hawaiʻi (Stamoulis & Freidlander, 2012). Although fishing is still permitted by law, Pelekane Bay has developed into a *de facto* marine protected area due to more limited access.

A seasonal effect among the three survey periods is most likely minimal relative to inter-annual differences in the overall fish abundance. For example, inter-annual variability of recruit abundance in Hawaiʻi is greater than the seasonal variability (Walsh, 1987). Lunar differences in recruitment and spawning periodicity have been reported for several species in Hawaiʻi (Walsh, 1987), but the three surveys used in the present analysis were conducted on multiple days with varying moon phases within each year. The potential effect of lunar phase on overall fish abundances were averaged and not biased towards new or full moon when recruitment and spawning are reported to occur for some species.

Results of the extensive studies by Storlazzi et al. (2013) and DeMartini et al. (2013) indicated that the turbidity, sediment cover and sediment accumulation rate are highest near the sediment source (stream mouth) and decrease on the reef with increasing distance from the stream mouth. Our study is in agreement with these observations. Biotic factors show an inverse relationship to this sediment pattern with the lowest rugosity, coral cover, coral richness, fish abundance, fish diversity, and evenness increasing with distance from the stream mouth.

Pelekane Bay has a long history of chronic land-based influences including sedimentation and resuspension which has affected coral reef recovery. Substantial sediment accumulation between 1928 and 2011 has occurred in Pelekane Bay (Storlazzi et al., 2013). Comparison of bathymetry over this time period revealed that 22,489 to 37,483 m^-3^ of sediment was deposited that resulted in a shoaling of 0.41 to 0.61 m during this time interval. Nevertheless natural resilience of reef ecosystems can facilitate recovery (Nyström & Folke, 2001). Full recovery to pre-disturbance levels may be an extended process, requiring many more decades. Even though the reefs have been damaged, our data show that further decline can be stopped and recovery can begin once stressors are reduced. Such damaged reefs may be prime candidates for restoration activities because at this point on the degradation curve a slight improvement in the environment may result in a greater improvement in coral and fish assemblages than might be observed from similar restorative effort on a mildly stressed reef. Our conclusion is that watershed restoration projects, reduced fishing pressure, and increases in marine protected areas in adjacent regions have allowed for partial recovery of fish populations since the Tissot (1998) surveys.

The community structure of the Pelekane Bay reef over the past two centuries apparently has changed in a manner that results in tolerance resistance to severe impacts including storm events and land-based sedimentation. Results of this survey show that the Pelekane Bay reef has the ability to absorb severe disturbance while continuing to maintain functional capacities. Factors that can affect reef resilience include improved water and substrate quality (Wolanski, Richmond & McCook, 2004), herbivore abundance, stable coral cover, and species and habitat diversity (McClanahan et al., 2012). These factors have all improved since the previous survey. Recent change in the reef community of Pelekane Bay exemplified the positive effects of an integrated approach of watershed management and acute wave disturbances on mitigating local human impacts.

The long-term data set that now exists for Pelekane Bay will be valuable in the future for continued assessment of reef community response to environmental change and improved management strategies. Continued monitoring and expansion of the original dataset will allow evaluation of relationships between abiotic and biotic factors. These data can be used to examine ecological trends and patterns in response to human and natural impact.
